# Importance of Media Composition and Explant Type in *Cannabis sativa* Tissue Culture

**DOI:** 10.3390/plants13182544

**Published:** 2024-09-10

**Authors:** Rekhamani Das, Tobias Kretzschmar, Jos C. Mieog

**Affiliations:** Faculty of Science and Engineering, Southern Cross University, Lismore, NSW 2480, Australia; tobias.kretzschmar@scu.edu.au (T.K.); jos.mieog@scu.edu.au (J.C.M.)

**Keywords:** *Cannabis* tissue culture, *Cannabis sativa*, micropropagation, shoot regeneration, vitrification

## Abstract

Producing uniform *Cannabis sativa (Cannabis)* for medicinal/recreational flower production through sexual propagation has been problematic, leading to dominance of clonal propagation from “mother plants” in the cannabinoid industry, which also faces significant limitations. *Cannabis* tissue culture (TC) methods have been developed to overcome these challenges, but the long-term health and maintenance of *Cannabis* explants in TC have been largely overlooked in previous studies. The current study focused on the development of an efficient and optimized micropropagation protocol covering the entire process, with a specific focus on the health and performance in the multiplication stage. Multiplication media were formulated hormone-free to avoid longer-term vitrification issues, resulting in single-main-shoot cultures rather than multiple-shoot cultures. This instigated the use of stage II explant types different from the standard shoot tips previously used for multiple shoot cultures. Multiplication media were further improved from the basal salt composition via nitrogen and calcium additives. The optimized protocol was used on eight diverse *Cannabis* cultivars to test its applicability across various genetic backgrounds. Results indicated that the protocol was effective for conservation purposes across all cultivars and achieved good long-term multiplication rates for some but not all. The outcomes of this study mark a significant stride towards an efficient *Cannabis* TC methodology ready for more comprehensive industrial applications.

## 1. Introduction

Global *Cannabis sativa* (*Cannabis*) markets have grown and matured in recent years as an increasing number of countries have legalized *Cannabis* for medicinal and/or recreational purposes as well as industrial uses. For instance, *Cannabis* has been legalized for medicinal purposes in various U.S. states, and CBD from industrial hemp is legal country-wide since the 2018 Farm Bill, while Canada and Thailand legalized it for both medicinal and recreational uses in 2018 and 2023, respectively [1,2,3,4]. Similarly, Germany ended *Cannabis* prohibition in 2021, becoming the second G7 nation to do so [5] after Canada, and since April 2024, a legal framework for recreational use has been implemented. In Australia, cultivation of *Cannabis* for cannabinoid production has been regulated at the federal level by the Office of Drug Control (ODC) since 2016, while industrial hemp cultivation for seed and fiber (thus excluding CBD and terpene production) has been regulated at the state level since 2008 [6].

The medicinal *Cannabis* industry depends on high-quality cannabinoid-rich products derived from female-only cultivation, and trimmed flower buds rather than extracts are the preferred product for end use [7]. To reliably achieve the required quality, production plants need a high level of uniformity. However, *Cannabis* cultivars are dioecious and highly heterozygous plants, severely complicating uniform propagation from seed. While feminized seed is available, these are still not true to type, resulting in inconsistent performance and product quality. Consequently, the *Cannabis* industry largely depends on clonal propagation, predominantly achieved via striking cuttings from mother plants. Even large-scale *Cannabis* producers still rely on this traditional and effort-intense propagation method, where mother plants, which are maintained in a perpetual vegetative state, typically take up around 15% of the growth space [8]. In addition to being inefficient, this cultivation method exposes the industry to risks of potential loss of germplasm and production capacity due to disease and pest outbreaks. Exacerbating detrimental effects is the decline in vigor among mother stocks over time due to the accumulation of somatic mutations leading to genetic mosaicism [9] and telomere shortening, requiring the periodical refreshment of the mother stocks.

To address these production bottlenecks, *Cannabis* growers are seeking biosecure and space-efficient clonal propagation techniques and germplasm conservation strategies. Micropropagation presents a promising approach compared to the traditional rooted cuttings method for the *Cannabis* industry [10], as it has the potential to efficiently generate large quantities of disease-free, genetically uniform plants [11]. Additionally, it provides pathways for long-term conservation. Micropropagation is a plant tissue culture (TC) technique for multiplying plants rapidly by culturing plant tissues or cells in vitro [12] and has been successfully industrialized for multiple plant-based industries, including horticulture (e.g., orchids and bananas), agriculture (e.g., potatoes and sugarcane), forestry for timber trees, and high-value medicinal plants [13,14,15]. It is able to achieve rapid clonal multiplication by utilizing either the direct or indirect organogenesis pathways [16]. Direct organogenesis is the process where plant organs are formed directly from explanted tissues, while indirect organogenesis involves the formation of organs through an intermediate stage where de-differentiated cells (callus) are initiated and amplified before they are regenerated into full plants [17].

The process of micropropagation is divided into four main stages and one preparation stage.

Pre-culture preparation—This stage involves the selection and preparation of a mother plant (source of explant collection) for TC. The health and quality of the mother plant greatly affect the success rate of culture establishment.

Stage I—Initiation of culture: In this stage the explants (typically meristems, buds, leaves, or other tissues) are collected, sterilized and initiated on a nutrient media with Plant Growth Regulators (PGR). The main objective is to establish aseptic cultures.

Stage II—Multiplication: The main goal in this stage is to grow the explant, into multiple shoots, commonly achieved by manipulating the balance of PGRs (between cytokinin and auxin) in the culture medium. These shoots are then separately subcultured under the same conditions. By repeating this process, exponential multiplication over multiple subcultures can be achieved.

Stage III—Rooting: The shoots regenerated from the multiplication stage are cultured on rooting media, characterized by different auxin levels compared to previous stages, in order to stimulate root growth and obtain rooted plantlets.

Stage IV—Acclimatization and Transfer: In the final stage of micropropagation, the rooted TC plantlets are transferred from the in vitro environment to an external environment via gradual/stepwise acclimatization. The rooted plantlets are initially acclimatized in a controlled environment like a greenhouse, then moved to the natural/production environment.

The recent liberalization of laws and the expansion of *Cannabis* markets have coincided with a surge in *Cannabis* research, including *Cannabis* TC. This field has seen significant advancements in developing protocols for direct organogenesis, with a focus on different explant types and method optimizations. Key studies in this area have centered on direct organogenesis from shoot tip and nodal explants, providing valuable protocols for these specific explant types [18,19]. A study by Hesami and Adamek [20] found that explants from the basal and near-basal parts of high-cannabinoid *Cannabis* cultivars exhibited superior multiplication and rejuvenation properties in tissue culture compared to the explants collected from the apical and middle parts of the source plant, highlighting the importance of explant selection. Kodym and Leeb [21] developed an alternative in vitro system mimicking nursery beds using photoautotrophic micropropagation, which contributed significantly to the understanding of direct rhizogenesis essential for plantlet development. Conservation of *Cannabis* germplasm was also carried out using cryopreservation and synthetic seed production using in vitro explants (nodal and shoot tip) [22,23,24]. Adaptations such as the reduction in the subculture period and increasing the size of the explant for greater efficiency further highlight the progress made in this field [25].

Despite this progress, *Cannabis* TC continues to face several challenges that hinder its commercial implementation. First, it is recognized that long-term culture of *Cannabis* in the multiplication stage can lead to vitrification (due to the gelling agent, PGR, and/or media composition [26], and drying or browning of leaves, collectively complicating sustained multiplication efforts [10,27,28]. Notably, previous reports frequently neglect long-term explant health and maintenance in TC, a gap highlighted by the lack of data on Stage II outcomes. Moreover, there is a discrepancy in terms of multiplication rate (average number of explants suitable for the next subculture produced per explant) reported, with some reporting a high number of multiple shoots in freshly initiated Stage I explants, [11,21,28,29], while others observed a low multiplication rate even when using the same protocol [25,30]. Finally, *Cannabis* is highly diverse both genetically and phenotypically; however, TC protocols have typically only been applied to a single or limited number of varieties, making it challenging to assess the broader-scale applicability of the current TC protocols across genetically distinct drug-type and hemp-type varieties [31]. Collectively, these concerns highlight the necessity for additional research in *Cannabis* TC, particularly for the multiplication stage (stage II), before commercial implementation can be considered.

This study aimed to develop an efficient and optimized *Cannabis* micropropagation protocol, with a specific focus on producing consistent and sufficiently high multiplication rates required for commercial adoption while maintaining vigor and health over extended periods. Additionally, the applicability of the optimized protocol was tested across a range of genetically diverse varieties. To achieve this, four type I explants (collected from the mother plant ex vitro) of a high-CBD drug-type variety were tested in four different initiation media. Successful cultures provided the five different types of stage II explants (collected from TC plantlets in vitro), which were tested across a wide range of (modified) multiplication media. The final step involved testing of the applicability of an optimized protocol on eight distinct varieties, including both drug-type and industrial hemp, including several rounds of stage II multiplication.

## 2. Results

### 2.1. Initiation Trials

#### 2.1.1. Surface-Sterilization

Fungal contamination (Figure A2a,b) on stage I explants was observed to manifest between the fourth- and fourteenth-day post-inoculation, and bacterial contamination (Figure A2c,d) appeared after 2–3 weeks in culture, resulting in substantial attrition rates. Without carbendazim, the shortest exposure time (10 min) to 1% sodium hypochlorite (NaOCl) treatment alone resulted in 100% contamination, whereas prolonged exposures (45 min or more) caused 75% or more tissue death (Table 1). Carbendazim treatment at 0.5 or 1 gm/L significantly reduced fungal contamination; however, it also significantly increased tissue death and reduced the viability of the explants, with the number of suitable explants for subculture dropping at 30 min 15 NaOCl treatment from 55% with no carbendazim to 10% in 1 gm/L carbendazim. Thus, due to these detrimental effects on tissue health, carbendazim treatment was not included in the selected sterilization procedure. The 1% NaOCl treatment for 30 min without carbendazim significantly reduced fungal contamination compared to shorter exposure times, while showing significantly less tissue death than longer exposures or exposures including carbendazim, leading to 55% of the explants being suitable for further TC (Table 1). Thus, the 30 min without carbendazim treatment was used as a sterilization treatment in an active subsequent experiment.

#### 2.1.2. Stage I Explant Type

Fungal contamination was observed to be significantly less in meristems and microshoot tips compared to shoot tips and nodal explants (Table 2). Bacterial contamination appeared to be higher for nodal explants (40% bacterial) and shoot tips (25% bacterial) compared to microshoot tips and meristems. Similarly, meristem explants appeared to have higher vitrification and stronger callus growth than microshoot tips. As the microshoot tip explant showed significantly lower contamination than the shoot tip and nodal explants, and appeared to have lower vitrification than the meristem, microshoot tip explants were selected for all subsequent culture establishments.

#### 2.1.3. Initiation Media Comparison

The microshoot tip explants in Initiation Media 3 (IM3), comprising Murashige and Skoog (MS) media [32,33] with 0.48 mg/L meta-Topolin (mT) [11], exhibited the best growth with rapid increases in size and length, and IM3 was selected as the initiation media for all subsequent culture establishments (Table 3). The microshoot tip explants in IM1, MS media with 0.11 mg/L Thidiazuron (TDZ) [29], exhibited stunted growth with dark green leaves, and the microshoot tip explants in IM2, MS media with 0.2 mg/L TDZ and 0.1 mg/L 1-naphthaleneacetic acid (NAA) [34], appeared more prone to vitrification and callus. The same explant type in IM4, Driver and Kuniyuki Walnut (DKW) [35] media with 0.11 mg/L TDZ [31], showed light green leaves and media browning near the base of the explant, indicating unhealthy explants.

### 2.2. Multiplication Trials

#### 2.2.1. Media Trial 0 (MT0)

**Figure 1:**
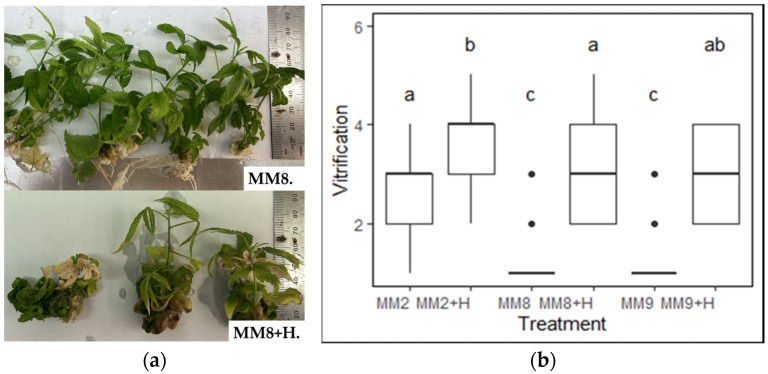
(**a**) The media comparison without (MM8) and with (MM8 + H) 0.5 mg/L meta-topolin. (**b**) Effect of different media compositions on vitrification (N = 15 × 3). The means were compared using Dunn’s test with a significance level of adjusted *p*-value < 0.05, significant differences are indicated by different letters for each treatment.

The effect of PGRs constitutes a key aspect of the multiplication (Stage II), as it is added to encourage the growth of multiple shoots from a single explant. In this experiment, the explants from IM3 were subcultured in six different media to assess the effect of the presence and absence of meta-topolin (mT) based on the vitrification level, a potential negative consequence (Figure 1). It was observed that the media with 0.5 mg/L meta-topolin (MM2 + H, MM8 + H and MM9 + H) showed significantly higher rates of vitrification compared to their PGR-free counterparts MM2, MM8, and MM9, respectively. The control group (MM2), despite having no PGRs, displayed a higher level of vitrification compared to MM8 and MM9.

#### 2.2.2. Media Trial 1 (MT1)

**Figure 2:**
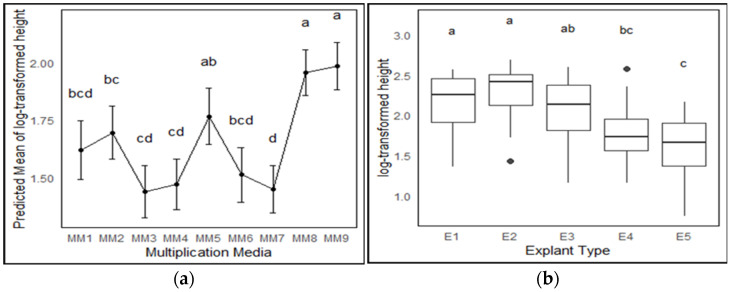
Effect of different media modifications and explant types on height (N = 15 × 3). (**a**) Height variation for predicted mean of all treatments without explant-type effect. (**b**) Effect of explant type (E1—primary shoot tips usually have many nodes with shorter internodal segments, E2—secondary shoot tips usually have fewer nodes with long internodal segments, E3—secondary shoot tip with primary node, E4—small secondary shoot tip with primary node, and E5—primary nodes with or without emerging bud) on height in MM8 and MM9 media in Media Trial 1. The means were compared using Tukey’s honest significant difference with *p* < 0.05, significant differences are indicated by different letters for each treatment.

**Figure 3:**
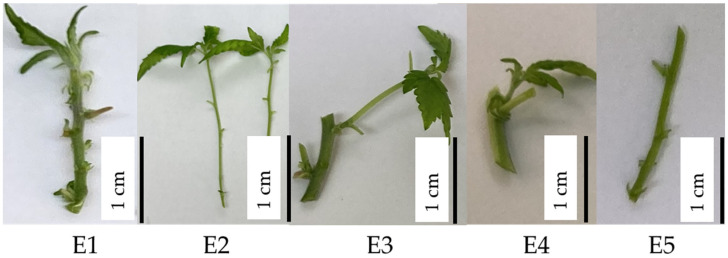
Different types of stage II explant types used in multiplication trials. E1—primary shoot tips usually have many nodes with shorter internodal segments, E2—secondary shoot tips usually have fewer nodes with long internodal segments, E3—secondary shoot tip with primary node, E4—small secondary shoot tip with primary node, and E5—primary nodes with or without emerging bud.

To analyze the effect of media composition and phase 2 explant type in the multiplication stage, the performance of five different phase 2 explant types was assessed on 11 different media modifications. It was observed that the explants in treatments with DKW (MM10) [35] and a combination of ½ DKW and ½ MS (MM11) [32] exhibited signs of severe distress 2–3 weeks after inoculation, including the presence of light green or bleached leaves, stunted growth or no growth, and a notable browning of the media near the bases of the explants. As they ultimately led to tissue death, MM10 and MM11 were discontinued shortly after the preliminary testing stage. The log-transformed data of height showed near-normal distribution (even though it failed the Shapiro–Wilk test). The predicted treatment height means (log-transformed) showed that the explants in MM8 and MM9 were statistically the same and superior to all other media composition except MM5, which showed comparable results to MM1, MM2, and MM6 (Figure 2a). Thus, data for MM8 and MM9 were pooled to assess the effect of phase 2 explant types (Figure 3). Height data exhibited a decrease from E1 to E5 (Figure 2b), with E1 and E2 showing significant differences from E4 and E5, whereas E3 did not exhibit significant differences from the E1, E2, and E4 explant types.

#### 2.2.3. Media Trial 2 (MT2)

**Figure 4:**
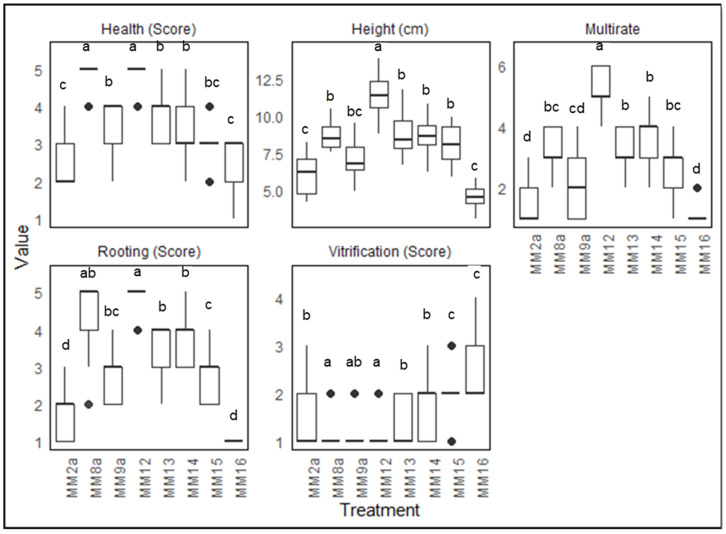
Effect of different media compositions on health, height, multiplication rate, rooting, and vitrification (N = 15 × 3). The means were compared using Dunn’s test with a significance level of adjusted *p*-value < 0.05; significant differences are indicated by different letters for each treatment.

In an attempt to further optimize the media, the two best-performing phase 2 explant types (E1 and E2—pooled for analysis) were tested against additional media formulations based on the two best performing media in MT1 (MM8 and MM9) (Figure 4). MM8 and MM12 showed the healthiest explants, followed by MM9, MM13, and MM14, and the rest showed reasonably healthy explants. For height and multiplication rate, MM12 outperformed the rest of the media compositions. MM8 and MM12 showed better results for rooting. In the indexing of the media, it was observed that MM12 outperformed the other treatments, with M8 ranked second (Table 4).

A correlation dot-plot was used to visualize the correlations among the traits measured for MT2 (Figure A3). Vitrification was negatively correlated with height, health, rooting, and multiplication rate, indicating a tendency for these parameters to decrease as vitrification increased. In contrast, positive correlations were observed between health, height, multiplication rate, and rooting, with height and multiplication rate being very highly correlated.

#### 2.2.4. Losses Due to Contamination in Multiplication

Endophytic contamination frequently appeared in stage II explants during the multiplication stage (stage II—3 to 4 weeks), causing loss of culture (Figure A2e,f), and also during root development (Figure A2g,h). This led to the reduction in the multiplication rate during the multiplication stage.

### 2.3. Acclimatization

Most of the explants rooted in the multiplication media (Figure A4) and showed a 90% survival rate (16 plants out of 18 transferred) when transferred to coco peat plugs. The plants in coco peat showed normal morphological growth. After successful acclimatization, they were successfully transferred to a greenhouse.

### 2.4. Applicability of the Optimized Protocol on Eight Diverse Varieties

Loss during the initiation stage was high at 90–95% for all varieties, except for IPK-CAN-44 (~75%) (Table 5). The multiplication rate for each subculture varied across the different subcultures and varieties, with the rate remaining within a range of 1 to 4. In the next subculture, the multiplication rate dropped to 1 to 3. The rooted explants after the three-multiplication round were successfully acclimatized and transferred to a greenhouse.

## 3. Discussion

### 3.1. Surface Sterilization

The results of this study highlighted the fine balance between effectiveness of sterilization conditions and preserving tissue health when performing surface sterilizations. When using bleach alone, a 30 min 1% NaOCl treatment was identified as optimal, with longer treatments causing significantly higher mortality and shorter treatments resulting in too high contamination (Table 1). A 10 min treatment at 1.5, 3.0, or 4.5% NaOCl concentrations was reported to be effective for surface disinfecting explants collected from clean and pest-free growth chambers of *Cannabis* plants with low endophytic microbes; however, all failed to remove contamination in explant-collected *Cannabis* plants grown in a greenhouse [36]. While for species such as *Sideritis Raeseri Boiss*, *Heldr. Subsp. Raeseri,* and *Peperomia pellucida*, carbendazim was found to be suitable for surface sterilization and media incorporation [37,38], in *Cannabis*, carbendazim was found to markedly increase explant mortality already at a concentration of 0.5 gm/L, with higher mortality rates at higher concentrations (Table 1). This might be due to its inhibitory effects on metabolic processes, as treatments with carbendazim had considerably higher tissue damage than treatments without it. Similar antagonistic effects were observed in tobacco plants when carbendazim was used in excess, which inhibited phenolic metabolism [39].

Although surface sterilization was able to markedly reduce the contamination, it failed to eliminate the *Cannabis* endophytes. *Cannabis* endophytes are believed to have the potential to enhance *Cannabis* growth and cannabinoid yields, though the details of their interaction are not fully understood [40]. However, endophytes also have been identified as likely attributing to a resurgence of contamination observed several weeks after an aseptic culture appears to have been established [41]. As it was found that endophytes could not be removed via surface sterilization, they likely need to be managed via explant type selection and/or subculturing approaches.

### 3.2. Initiation

Among the four initiation media tested, IM3 was selected as it appeared to produce explants with rapid and sustained growth. Microshoot tip explants (less than 0.5 cm in size and near meristematic tissue) exhibited the best overall results, characterized by very low levels of contamination, vitrification, and callus formation (Table 2). The low levels of contamination might be due to a low level of endophytes in actively growing shoot tips, as previously reported in *Oryza sativa* [42]. Shoot tip and nodal explants were clearly more affected by fungal and bacterial contamination (Table 2), possibly as a result of a heavier endophytic load as observed previously [25,28,43]. Meristematic explants, while free from contamination, showed increased vitrification and callus formation, potentially causing unwanted soma-clonal variation, likely due to their high metabolic activity and mostly undifferentiated state [44,45,46,47]. Thus, it is advised to use microshoot tip explants for initiation stage 1 TC. However, a decline in health was observed after 2–3 subculture rounds in this medium, characterized by decreased growth and increased vitrification, as evidenced by light green, translucent, and brittle leaves. Similar observations were reported by Lubell-Brand and Kurtz [8]. This decline in health over time likely indicated excessive humidity and an imbalance between the supplied and endogenous PGRs that exacerbated over time, suggesting that a different media composition and PGR formulation for the multiplication stage (Stage II) would be of benefit.

### 3.3. PGRs and Vitrification

The use of the PGR meta-topolin in stage II multiplication media was found to negatively affect *Cannabis* TC by significantly increasing vitrification (Figure 1), which is in contrast to other crops like bananas, ornamental trees, and coconuts where they enhanced growth and multiplication [12,48,49,50]. This vitrification was likely the result of an excessive presence of cytokinins in the media formulated with meta-topolin, which, especially when combined with ammonium, has been found to contribute to vitrification in *Cannabis*, causing leaf narrowing, leaf curling, and stunted growth [25,43]. Previous studies mostly relied on the addition of PGRs to induce multiple shoot cultures, where each shoot could be subcultured to achieve a high multiplication rate in an effort to replicate the success seen in other crops [36,51,52]. Similarly, the PGR Thidiazuron (TDZ) increased fresh weights, shoot numbers, and lateral nodes, but it also increased vitrification and callus formation, leading to physiological abnormalities and multiplication losses of up to 90% [36,53]. Notably, the control group (MM2), despite having no meta-topolin, displayed a higher level of vitrification compared to MM8 and MM9, indicating that while PGRs contribute to vitrification, they are not the sole influencing factor.

### 3.4. Multiplication Via Single Main Shoot Cultures

For multiplication, the exclusion of PGRs (cytokinins) in the media to reduce/avoid vitrification meant that, instead of a multiple shoot culture, the aim was to grow a single, high-quality main shoot. Consequently, sections of the main shoot would need to serve as explants for subsequent subcultures to achieve a multiplication rate greater than 1. To manage growth constraints imposed by the container’s height, the subculture period was consistently set at four weeks.

In the multiplication stage, primary and secondary shoot tips (E1 and E2) performed significantly better than other types (E4 and E5), with no differences between E1 and E2. (Figure 2). E3 was not significantly different from either group. This enhanced performance of shoot tips close to the top might be attributed to the high levels of endogenous PGRs present in the actively growing parts of these explants. Additionally, the improved outcomes could also be linked to apical dominance exhibited by in vitro *Cannabis* explants [25,30,43].

### 3.5. Multiplication Media

MS media was selected as the base for the multiplication media as it has been widely used for *Cannabis* micropropagation [11,25,54]. However, unmodified MS media (MM2) in absence of PGRs caused increased vitrification and reduced performance (Figure 2). Inadequate supply of calcium in media has been reported to lead to accumulation of phenolic compounds and cell death, while the level of calcium in the media also directly affects auxin transport and ethylene production and could encourage the shoot tip necrosis and vitrification [55]. It is thus likely that MS media alone is not able to fulfill the nutritional requirements of a vigorous feeder like *Cannabis*. Lubell-Brand, Kurtz [8] reported that the addition of 500 mg/L ammonium nitrate in modified shoot multiplication media with vitamins and a mixture containing calcium chloride (anhydrous), magnesium sulfate (anhydrous), and monobasic potassium phosphate (also called mesos), effectively doubled the multiplication rate by invigorating the *Cannabis* explants for at least 12 weeks following the initiation step. The addition of 1 gm/L calcium gluconate to the TC media was also reported to efficiently control shoot necrosis in primocane-fruiting raspberry micropropagation, where in vitro shoot necrosis was a major problem [56]. In this study, the addition of calcium nitrate alone enhanced growth and height, while calcium gluconate alone improved explant quality by decreasing vitrification; however, their combined use yielded the optimal results (Figure 4).

In this study, ferric-sodium EDDHA (Fe-EDDHA) was found ineffective in increasing shoot induction and multiplication or improving the quality of the *Cannabis* explants. In contrast, the explants on media containing Fe-EDDHA were observed to perform poorly compared to those without it. Although the use of an Fe-EDDHA with BAP was reported to increase shoot induction and multiplication in species such as *Baptisia australis*, *Rubus fruticosus,* and *Hibiscus rosa-sinensis* by either neutrally or negatively influencing the strong stimulus of BAP on the induction of apical buds and nodal sections [57,58,59], this was not observed in our study.

The agar concentration from 7 gm/L in MT1 was increased to 9.5 gm/L in MT2 to address the issue of high vitrification through the reduction in humidity inside the container. A similar addition was reported to improve the health of *Cannabis* TC and reduce vitrification [60]. Although not tested separately, the best performing media had the increased agar concentration suggesting that the higher agar concentration, may be of benefit.

### 3.6. Traits to Measure Explant Quality

The correlation among observed parameters in MT2 showed that height, rooting, health, and multiplication rate were positively correlated with each other and negatively correlated with vitrification (Figure A3). Height showed positive correlation with health (0.69), rooting (0.82), multiplication rate (0.87), and negative correlation with vitrification (−0.51). Therefore, it proved to be a reliable indicator and could be used as the primary selection trait, as was performed in MT1. Vitrification showed negative co-relation with health (−0.77), rooting (−0.61), and multiplication rate (−0.47).

### 3.7. Applicability

Eight diverse varieties were tested with the best-performing initiation method (Section 4.3.1) and multiplication method (Section 4.3.2). Losses during initiation were higher than expected at 45% (Table 5). The low survival rates during initiation could be attributed to high initial contamination from the presence of numerous endophytes typical of unimproved germplasm. The contamination-free explants were successfully maintained through three subcultures in MM12 media. The different varieties showed different multiplication rates in the same multiplication medium ranging from 1 to 4 in the second subculture to 1 to 3 in the third subculture. As a comparison, the multiplication rate of the commercial CBD line used for the method development, which went through at least four subculture rounds before being used in the multiplication trials, showed a multiplication rate ranging between 4 and 6 per explant in MM12 media (Figure 4). This variation in multiplication rate in different varieties illustrates the genotype-dependent nature of *Cannabis* tissue culture, which has also been reported by others [30]. The optimized protocol’s success in integrating all eight lines into tissue culture and being able to maintain them in the multiplication stage was considered a significant step forward. However, depending on the genotype, further increases in the multiplication rate may be crucial for successful commercial production. This study was primarily focused on the multiplication phase of micropropagation, and as such, it is limited in addressing the broader issues of vitrification and contamination. Further research is necessary to better understand these effects and their potential correlations with other factors.

### 3.8. Conclusions

The detailed investigation of the initiation and multiplication stages of *Cannabis* micropropagation yielded several significant findings. Bleach-based surface sterilization proved adequate for surface decontamination but was ineffective against endophytes. The selection of the microshoot tip as the phase 1 explant type significantly enhanced growth and helped reduce contamination and vitrification compared to most other explant types. The use of the PGR meta-topolin was found to be detrimental in multiplication (stage II) due to the increase in vitrification, necessitating a shift in focus from multiple shoot cultures to growing single main shoot cultures in this stage. The stage II explant types with shoot tips (E1 and E2) produced optimal results across multiple subcultures. The inclusion of calcium nitrate and calcium gluconate in the multiplication media was determined to enhance the overall *Cannabis* TC outcome. This optimized protocol was successfully applied to eight different varieties, which demonstrated its effectiveness and adaptability across a diverse range of *Cannabis* genetics for conservation purposes, although genotype-dependency meant that further increases in the multiplication rate may be crucial for successful commercial production.

## 4. Materials and Methods

### 4.1. Genetic Resources and Mother Plant Cultivation

The preliminary initiation and multiplication trials were conducted with a high-CBD cultivar cultivated at the Cymra Life Science Northern Rivers campus (Northern Rivers, NSW, Australia), who were appropriately licensed to hold the material. Mature *Cannabis* mother plants growing in a greenhouse served as the explant source. The TCs at Cymra were maintained in a rack fitted with slimline seamless TC linkable SL9706-4/22 LED tube lights from SAL, Australia with ~3000 lux light intensity, growth room at temperature 25 ± 2 °C and 60% relative humidity.

The genotypes selected for variety trialing were mainly from Eurasian origins obtained from the “IPK genebank collection” of the Leibniz Institute of Plant Genetics and Crop Plant Research (Leibniz-Institut für Pflanzengenetik und Kulturpflanzenforschung, IPK—Germany) and were grown at the Southern Cross University Lismore campus (Lismore, NSW, Australia). The experimental work on the high THC *Cannabis* varieties at Southern Cross University (Lismore, Australia) was conducted under an authority granted to Prof. Bronwyn Barkla of Southern Cross University (SCU), issued by the New South Wales Ministry of Health (Australia). The experimental work on the low-THC industrial hemp varieties at Southern Cross University (Lismore, Australia) was conducted under hemp license number 52204 issued by the NSW Department of Primary Industries (Orange, Australia).

Fifty seeds from the varieties were germinated, and 20 seedlings were selected and grown in seed trays that served as the explant source. The plants were grown on premix potting soil mix with 70% coco, 30% perlite, and 3.4 g/L osmocoat exact 3–4 in a grow room. The TCs were maintained in a Versatile Environmental Test Chamber (Sanyo model MLR-350T) at temperature 25 + 2 °C and ~3000 lux.

### 4.2. Initiation

#### 4.2.1. Surface-Sterilization Trial

**Figure A1:**
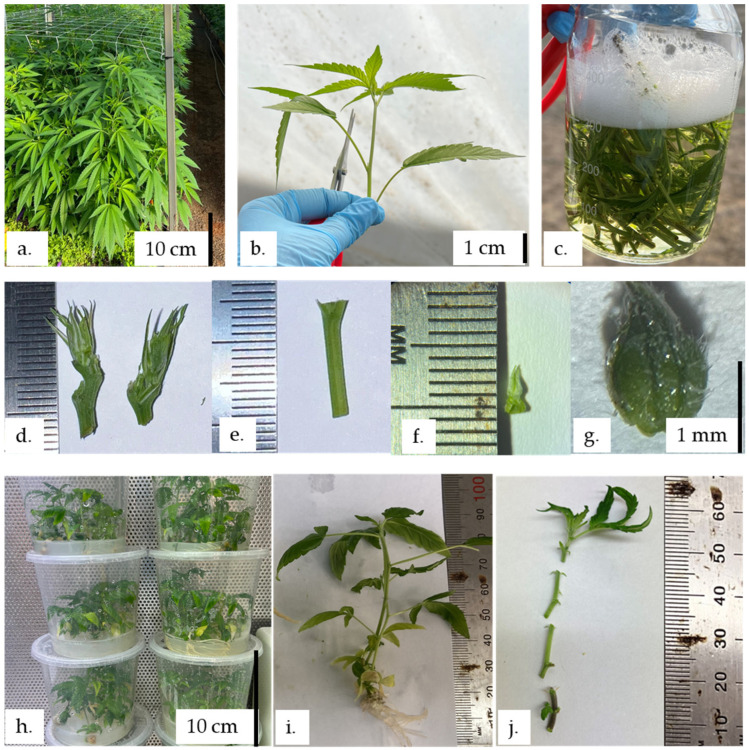
Tissue-culture images with different micro-propagules. Stage I explants: (**d**–**g**), Stage II explants: (**h**–**j**). (**a**) High-CBD cultivar mother plants in green house. (**b**) 6–8 cm shoot tip for tissue culture. (**c**) Surface sterilization treatment. (**d**) Shoot-tip (>0.5 cm). (**e**) Nodal (1 cm–0.5 cm). (**f**) Microshoot tip (<0.5 cm). (**g**) Meristem. (**h**) *Cannabis* culture. (**i**) *Cannabis* tissue culture plantlets. (**j**) Processed *Cannabis* plantlet.

For establishing the TC, phase 1 explants were taken from the mother plants (ex vitro) and surface-sterilized (Figure A1a–c) before being inoculated in initiation media. An initial surface-sterilization trial assessed the effectiveness of 1% NaOCl (premium chlorine bleach 4%) sterilization treatment, with and without carbendazim (a broad-spectrum fungicide), on reducing contamination and minimizing tissue death after four weeks post-initiation. Excised stage I explants (shoot tip and nodes) were washed in autoclave distilled water with a few drops of undiluted Tween-20 to remove the adhering dust particles. The washed explants were immersed in 1% NaOCl in a 1 L Schott bottle and rotated in a rotary shaker at 150 rpm for 10 to 60 min. Next, treated explants were washed 5 times with autoclaved distilled water. Selected explants were treated with 0.5 gm/L or 1 gm/L concentration of carbendazim for 15 min and washed as above before being trimmed and inoculated onto MS media with 0.5 mg/L meta-topolin. For each treatment group, twenty explants were used. Explants with contamination, tissue death, and suitable for subculture (stage II) were measured as binary traits (yes/no).

#### 4.2.2. Stage I Explant Type Used in Initiation Trial

**Figure 5:**
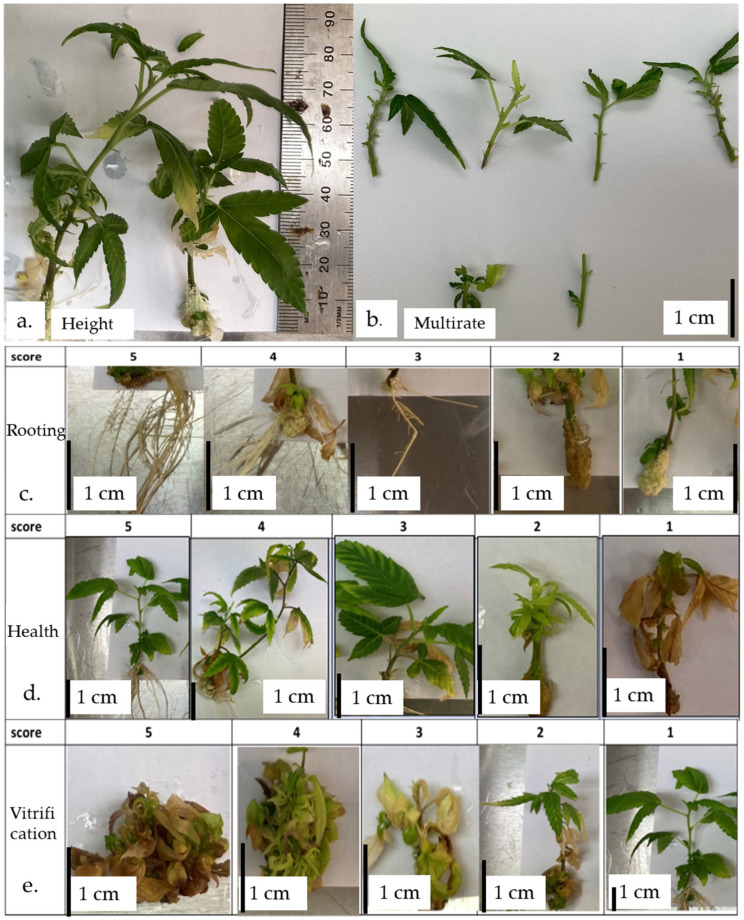
The traits measured in the multiplication trials (MT0-2). (**a**) plant height (cm), (**b**) multiplication rate (count), (**c**) plant rooting (score 5–1 based on the density of the roots from most densely rooted to no rooting), (**d**) plant health (score from 5 to 1, ranging from best to worst), and (**e**) vitrification (scored from 5 to 1, ranging from highly vitrified to no vitrification).

Four different types of Stage I explant types, meristem, microshoot tip, shoot tip, and nodal segments (Figure A1d–g), were surface-sterilized with a 30 min 1% NaOCl treatment and initiated on media as described under 4.2.1. Each Stage I explant type treatment consisted of 20 units (4 explant/700 mL container). Vitrification was recorded as presence (Figure 5e score 5 to 2) or absent (Figure 5e score 1). Contamination and vitrification of the explants were recorded 4 weeks after initiation as binary traits (yes/no).

#### 4.2.3. Stage II Explant Types Used in Multiplication Trials

After 4 weeks, the stage I explants from initiation media that had grown without showing any sign of contamination during initiation were subcultured in MS basal media with MS vitamin mix without hormone for another 2–3 subculture rounds to generate stage II explants needed to assess the influence of explant type on multiplication (Figure 2 and Figure 4). The stage II explants utilized in the multiplication trials were: E1—primary shoot tips usually characterized by multiple nodes and relatively short internodal segments; E2—secondary shoot tips usually characterized by fewer nodes and longer internodal segments; E3—secondary shoot tip with primary node; E4—small secondary shoot tip with primary node; and E5—primary nodes with or without emerging bud.

### 4.3. Media Composition

#### 4.3.1. Media Compositions Used in Initiation Trial

Stage I explant types as described for Section 4.2.2 were surface-sterilized with a 30 min 1% bleach treatment and initiated as described under Section 4.2.1. on four different media as listed in Table 6. Three initiation media were based on MS, while one was based on DKW. The media were prepared by making stock solutions. Stock solution A (all macronutrients), stock B (micronutrient), stock C (for iron), and stock D (vitamins) were obtained from ChemSupply, Australia. Micropropagation-grade agar from the PhytoTech lab, Australia, was used as a gelling agent. A total of 30 gm/L (3%) sucrose from ChemSupply, Australia, was added. The pH of the media was adjusted to 5.7–5.8 using 1 mM of NaOH and subsequently autoclaved for 20 min at 121 °C, and 18 psi. PGRs (NAA, mT, and TDZ) sourced from Phytotech, Australia, were filter sterilized using a millex-GP filter unit (sterile) from Sigma-Aldrich, St. Louis, MO, USA, and added to media after autoclaving. Media were prepared in 1 L Schott bottles and poured in 30 mL sterile initiation tubes in a laminar hood. Each media composition consisted of 20 replicates (1 explant/30 mL tubes, n = 20). Contamination and vitrification were recorded 4 weeks after initiation, similar to Section 4.2.2.

#### 4.3.2. Media Compositions Used in Multiplication Trials

The media compositions tested in multiplication trials included half-strength MS medium, standard MS medium, modified MS medium enhanced with the (0.1 mM = 45.32 mg/L) Fe-EDDHA, CAS No. 16455-61-1, instead of (0.1 mM = 37.26 mg/L) ferric-sodium EDTA, DKW medium, and a combination of ½ DKW and ½ MS media (Table 7). Modifications to the basal media involved calcium gluconate (CAS No. 66905-23-5), calcium nitrate (CAS No. 13477-34-4), and agar (CAS No. 9002-18-0) sourced from Phytotech Australia. The media preparation followed the same procedure as the initiation media and was poured into a 700 mL sterilized round plastic container in a laminar hood.

A preliminary multiplication media trial (MT0) was conducted to investigate the impact of plant growth regulators (PGRs) on the occurrence of vitrification. Three variations of MS media (MM2, MM8, and MM9) with and without 0.5 mg/L meta-topolin, the most widely used PGR for cannabis micropropagation [11,30,31,61,62], were tried (Table 7) on the different stage II explants as described in Section 4.2.3 using media sterilization as described in Section 4.3.1. Each media treatment consisted of 15 units (5 explants per container) and was repeated thrice (N = 45). The vitrification data were collected after 4 weeks of culture. Vitrification was scored on a scale of 5 to 1, from highly vitrified (5) to no vitrification (1) (Figure 5).

**Figure A2:**
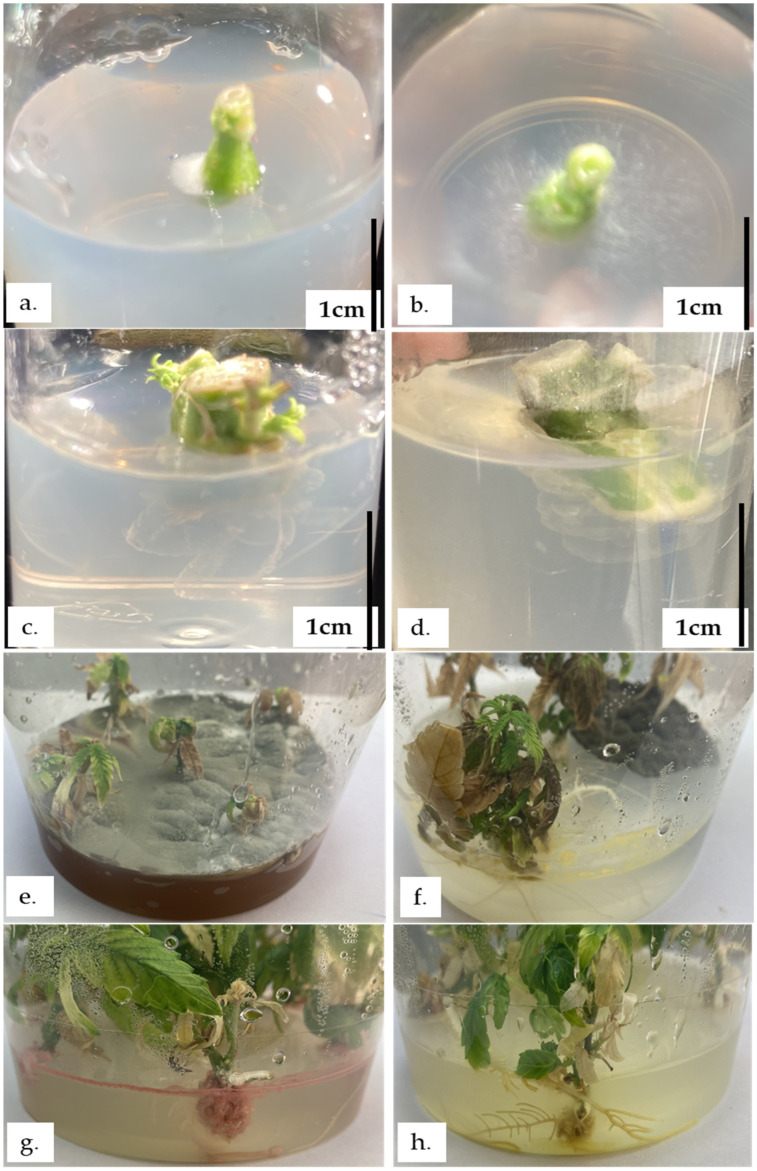
Fungal and bacterial contamination in different stages of *Cannabis* tissue culture. (**a**,**b**)—Explants fungal contamination in stage I. (**c**,**d**)—Explants showing bacterial contamination stage I. (**e**,**f**)—Explants showing different types of fungal and bacterial contamination Stage II. (**g**,**h**)—Explants showing endophytic bacterial contamination during root development in containers with a 10 cm base diameter.

**Figure A3:**
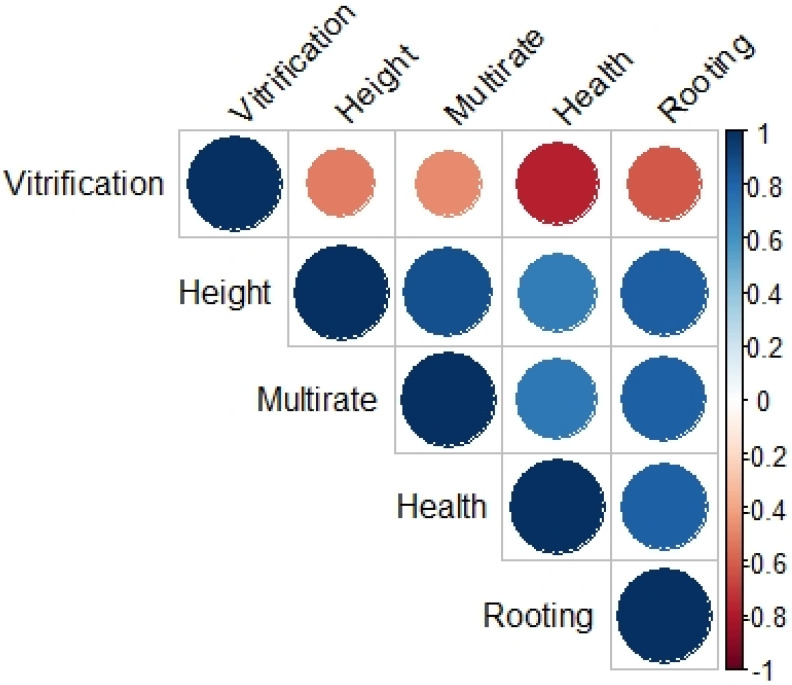
Dot-plot showing the average correlations of the MT2 measurement traits over all treatments and explant types. Negative correlations are shaded red, and positive correlations are shaded blue. The strength of the correlation is indicated by dot size and red or blue color saturation.

**Figure A4:**
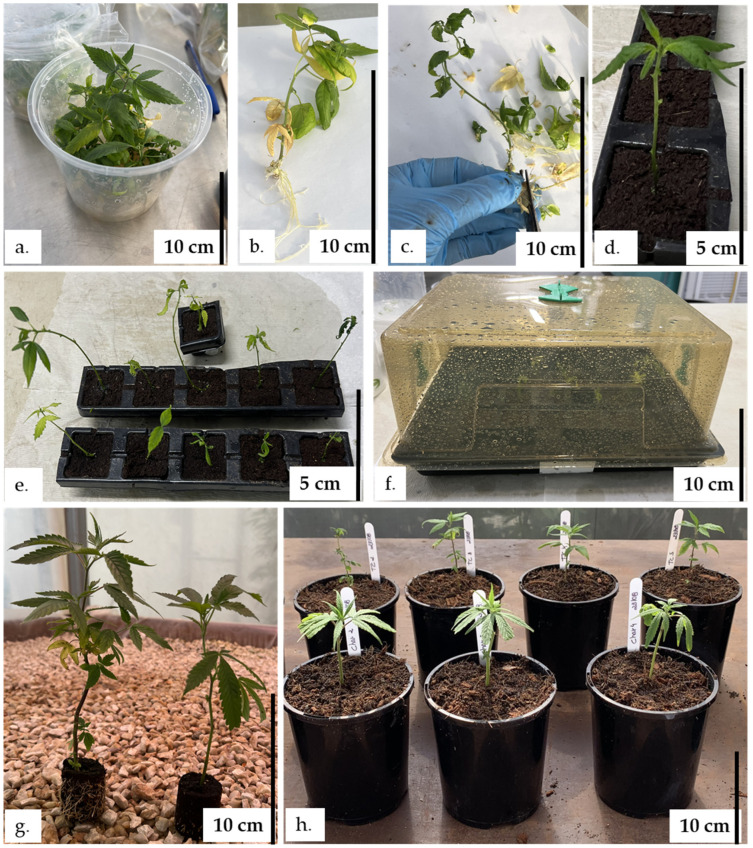
Deflasking and acclimatization of the *Cannabis* TC plantlets. (**a**)—*Cannabis* TC plantlet on media, (**b**)—TC plantlet, (**c**)—Trimming of the leaves and roots before placing in seed tray, (**d**)—planting in seed tray, (**e**)—TC generated plantlet ready for acclimatization, (**f**)—TC plantlets in mini greenhouse, (**g**)—rooted TC plantlet and cutting of Charlotte’s Angle, and (**h**)—rooted cuttings and acclimatized TC generated plants of high CBD cultivar potted in potting mix.

Multiplication media trial 1 (MT1) was conducted to assess the influence of stage II explant type and media composition on height. Five distinct types of stage II explants, as described in Section 4.3.1, were cultured on eleven different media (T1–T11) (Table 7). Explant heights were recorded (Figure A2a). Replication, media sterilization, culture maintenance, and data collection time were the same as for MT0.

The aim of multiplication media trial 2 (MT2) was to further optimize the best-performing media for the best-performing explant types as established by MT1. The E1 and E2 stage II explants were tested on modifications of MM8 and MM9 media compositions. Agar was increased from 7 gm/L to 9.5 gm/L, and the calcium gluconate and calcium nitrate compositions were modified to assess the effect of additional calcium and nitrogen in the media (Table 7). Explant height and vitrification were recorded the same as for MT1 and MT0, respectively. Rooting and health were assessed on a 5–1 score (Figure A2c,d)m and multiplication rate was recorded (number of explants from a single explant for the next subculture). Replication, media sterilization, culture maintenance, and data collection time were the same as for MT0.

#### 4.3.3. Indexing the Media Based on MT2 Results

The media compositions were ranked according to the index:

Index = Height + Multiplication Rate+ Health + Rooting − Vitrification

Height, multiplication rate, health, and rooting were considered to be positively correlated to TC success and were scored as follows: a = 5, b = 4, c = 3, d = 2, e–g = 1. Vitrification was considered detrimental to TC success and score as: a = 1, b = 2, c = 3, d = 4, e–g = 5.

### 4.4. Acclimatization

Stage II explants started rooting after 2–4 weeks when transferred in fresh hormone free multiplication media. Well-rooted plantlets (6–8 cm) were deflasked (taken out from the medium) after 4 weeks in multiplication media. The roots of the plantlet were treated with an antifungal (Pervicure fungicide from Crop Science Australia) solution, and excess agar was removed. After that, plantlets were transplanted onto coco peat plugs (Eazy Plug Coco Peat Propagation Tray from Hydrocentre Hydroponics, Australia) and kept covered in a mini greenhouse with movable air lid boxes. For the first weeks, the plantlets were sprayed with water every day to keep the humidity level high. After 10 days, slowly the movable air lid was adjusted to allow the humidity to reduce, followed by no cover, and maintained in a greenhouse. Plants were then transplanted in small plastic pots and finally in bigger pots. One month after deflasking, the survival frequency of regenerated plants was recorded.

### 4.5. Compilation of the Optimized Protocol and Testing on Different Cannabis Varieties

The optimized protocol was tested on eight genetically diverse varieties, including both *Cannabis* (<1% THC) and hemp (>1% THC) (Table 5). Sixty explants were collected from 3-week-old plants. The stage 1 (microshoot tip) explants were surface sterilized using 1% NaOCl for 30 min from Section 4.2.1 and initiated in the initiation medium (IM3) from Section 4.3.1. After the 2–3 weeks post-initiation, the healthy, contamination-free stage II explants were subcultured in MM12 media without hormone from Section 4.3.2. The explants were consistently maintained in the same media across three subcultures, each separated by a four-week interval, through the process of transferring them into fresh media for each subculture stage. The rooted plantlets were deflasked four weeks after the last subculture and transferred into coco peat plug and maintained in a mini greenhouse for two weeks for acclimatization. The fully rooted plants were transplanted in a bigger pot before transferring them to a greenhouse.

### 4.6. Statistical Analysis

Data from all experiments were analyzed using R 4.1.0 (R-studio 23.03.0 build 386 © 2009–2023 Posit Software, PBC) and the packages (ggplot2, ggpubr, tidyverse, broom, agricolae, reshape2, emmeans, dplyr, dunn.test, corrplot, and Hmisc). All experimental data were collected at the individual explant level, which served as the units of replication in the study. For the initiation stage (stage I), where data were collected as a binary response (yes/no), data were analyzed using a generalized linear model with a binomial distribution with main effects compared using Tukey’s HSD at *p* < 0.05 for mean separation. For multiplication stage (stage II), in MT1, the height data were log transformed to achieve near-normal distribution based on Shapiro–Wilk Test and Q–Q (quantile–quantile) plot. A two-way ANOVA was performed with explant type and treatment specified as factors. The treatments were compared using predicted values to select the best-performing media. Then, the data for the best-performing media were pooled, and a one-way ANOVA was performed to establish the effect of explant type for these media. For MT2, non-transformed data of traits (height, health, vitrification, rooting, and multiplication rate) were evaluated using normality tests Shapiro–Wilk and Q–Q (quantile-quantile) plot, which revealed that traits including height did not follow a normal distribution. This necessitated using the Kruskal–Wallis test, succeeded by Dunn’s test for conducting multiple comparisons for MT2. The parameters were compared using a correlation dot plot.

## Figures and Tables

**Table 1:** (**a**) Effectiveness of different surface sterilization treatments using different 1% NaOCl exposure times and carbendazim concentrations (n = 20). (**b**) Main effects of the surface sterilization treatments experiment and statistical significance of the observed differences in contamination, tissue death, and number of explants suitable for subculture.

**(a)**																					
**Treatment**	**Carbendazim (gm/L)**	**1% NaOCl (minute)**	**n**	**Contamination**						**Tissue Death**						**Suitable for Subculture**					
				**n**			**%**			**n**			**%**			**n**			**%**		
T1	0	10	20	20			100.00			1			5.00			0			0.00		
T2	0	20	20	13			65.00			3			15.00			4			20.00		
T3	0	30	20	8			40.00			6			30.00			11			55.00		
T4	0	45	20	4			20.00			15			75.00			4			20.00		
T5	0	60	20	3			15.00			20			100.00			0			0.00		
T6	0.5	10	20	13			65.00			12			60.00			6			30.00		
T7	0.5	20	20	8			40.00			13			65.00			6			30.00		
T8	0.5	30	20	6			30.00			15			75.00			4			20.00		
T9	0.5	45	20	2			10.00			20			100.00			0			0.00		
T10	0.5	60	20	2			10.00			20			100.00			0			0.00		
T11	1	10	20	9			45.00			17			85.00			2			10.00		
T12	1	20	20	8			40.00			17			85.00			2			10.00		
T13	1	30	20	6			30.00			20			100.00			0			0.00		
T14	1	45	20	2			10.00			20			100.00			0			0.00		
T15	1	60	20	0			10.00			20			100.00			0			0.00		
**(b)**																					
**Main Effect**		**n**		**Contamination**						**Tissue Death**						**Suitable for Subculture**					
				**n**		**%**		CLD *		**n**		**%**		CLD *		**n**		**%**		CLD *	
Carbendazim																					
0		100		48		48.00		a		45		45.00		a		19		19.00		a	
0.5		100		31		31.00		b		80		80.00		b		16		16.00		a	
1		100		27		27.00		b		94		94.00		c		4		4.00		b	
NaOCl																					
10		60		42		70.00		a		30		50.00		a		8		13.33		a	
20		60		29		48.33		ab		33		55.00		a		12		20.00		a	
30		60		20		33.33		bc		41		68.33		a		15		25.00		a	
45		60		8		13.33		c		55		91.67		b		4		6.67		a	
60		60		7		11.67		cd		60		100.00		b		0		0.00		a	

* Mean separation in columns using Tukey’s honestly significant difference at *p* < 0.05, CLD = compact letter display.

**Table 2:** Analysis of concomitant response to different stage I explant types (n = 20).

Stage I Explant	Contamination Fungal (%)	Contamination Bacterial (%)	Callus (%)	Vitrification (%)
Meristem	0.00a ^1^	0.00a	60.00a	50.00a
Microshoot tip (<0.5 cm)	5.00a	5.00a	20.00a	20.00a
Shoot Tip (>0.5 cm)	50.00b	25.00a	20.00a	20.00a
Nodes (>0.5 cm)	85.00c	40.00a	35.00a	35.00a

^1^ Mean separation in columns using Tukey’s honestly significant difference at *p* < 0.05.

**Table 3:** Analysis of optimal response to different initiation media (IM) (n = 20).

Name	Size Increase (%)	Vitrification (%)	Callus (%)	Observation after 4 Weeks
IM1	70.00a ^1^	40.00a	45.00a	Leaves dark green, stunted growth with 3–4 multiple shoots
IM2	60.00a	65.00a	70.00a	Leaves green, callus, vitrification with single shoot
IM3	70.00a	35.00a	45.00a	Leaves light green, better growth with 2–3 multiple shoots
IM4	40.00a	35.00a	30.00a	Leaves light green, media turned brown tissue death with single shoot.

^1^ Mean separation in columns using Tukey’s honestly significant difference at *p* < 0.05.

**Table 4:** Indexing the media based on the observations in Figure 4. For height, multiplication rate, health, and rooting, 1 = high/better and 5 = low/worst. For vitrification, 1 = low vitrification and 5 = high vitrification.

Media	Height	Multiplication Rate	Health	Rooting	Vitrification	Total
	(+)	(+)	(+)	(+)	(−)	
MM2	2.00	2.00	3.00	2.00	2.00	7.00
MM8	4.00	3.50	5.00	4.50	1.00	16.00
MM9	3.50	2.50	4.00	3.50	1.50	12.00
MM12	5.00	5.00	5.00	5.00	1.00	19.00
MM13	4.00	4.00	4.00	4.00	2.00	14.00
MM14	4.00	4.00	4.00	4.00	2.00	14.00
MM15	4.00	3.50	3.50	3.00	3.00	11.00
MM16	3.00	2.00	3.00	2.00	3.00	7.00

**Table 5:** Different varieties tried for the optimized media from initiation and multiplication trials. Initiation number 20 repeated 3 times (n = 20 × 3). The 1st subculture in 3 weeks, the second and third subcultures in 4 week intervals.

IPK	Dominant Chemotype	Variety Name	Country of Origin	1st Subculture Survival Rate	2nd Subculture Multiplication Rate	3rd Subculture Multiplication Rate
CAN_100	>1% THC	Gelb	Unknown	0.05	4.00	1.50
CAN_20	>1% THC	Unknown	North Korea	0.07	3.80	1.20
CAN_36	>1% THC	Fibridia	Unknown	0.03	2.00	1.00
CAN_29	>1% THC	Unknown	Romania	0.05	1.70	1.20
CAN-44	<1% THC	Fibrimon	Germany	0.23	1.00	1.70
CAN-17	<1% THC	Unknown	Hungary	0.03	1.00	1.50
CAN-30	<1% THC	Bernburger	Germany	0.05	1.30	2.50
CAN-43	<1% THC	Hohenthürmer Gleichzeitig Reifender	Germany	0.02	3.00	1.70
Commercial high CBD line	<1% THC	Unknown	Netherlands	0.45	4.00	6.00

THC—Delta(9)-tetrahydrocannabinol.

**Table 6:** The 4 types of initiation media (IM) used for culture establishment.

Media Name	Base Media	Supplements	Gelling Agent	Publication
IM1	MS	0.11 mg/L TDZ	0.80% Type E agar	[29]
IM2	MS	0.20 mg/L TDZ + 0.1 mg/L NAA	0.68% agar	[34]
IM3	MS	0.48 mg/L mT	0.80% Type E agar	[11]
IM4	DKW	0.11 mg/L TDZ	0.70% agar	[31]

MS—Murashige and Skoog, DKW—Driver and Kuniyuki Walnut media, TDZ—Thidiazuron, NAA—1-Naphthaleneacetic acid, and mT—meta-topolin.

**Table 7:** The different media compositions used in the multiplication trials.

Media Name	Base Media	Supplements		Gelling Agent (agar)
		Calcium Nitrate (gm/L)	Calcium Gluconate (gm/L)	
MM1	1/2MS	nil	nil	0.80%
MM2	MS	nil	nil	0.80%
MM3	Modified MS for iron	nil	nil	0.80%
MM4	1/2MS	nil	1.35	0.80%
MM5	MS	nil	1.35	0.80%
MM6	Modified MS for iron	nil	1.35	0.80%
MM7	1/2MS	0.71	nil	0.80%
MM8	MS	0.71	nil	0.80%
MM9	Modified MS for iron	0.71	nil	0.80%
MM10	DKW	nil	nil	0.80%
MM11	1/2DKW and 1/2MS	nil	nil	0.80%
MM2a	MS	nil	nil	0.95%
MM8a	MS	0.71	nil	0.95%
MM9a	Modified MS for iron	0.71	nil	0.95%
MM12	MS	0.71	1.35	0.95%
MM13	Modified MS for iron	0.71	1.35	0.95%
MM14	MS	1.42	nil	0.95%
MM15	Modified MS for iron	1.42	nil	0.95%
MM16	MS	1.42	1.35	0.95%

MS—Murashige and Skoog, Modified MS for iron—MS media with alternative iron source Fe-EDDHA instead of ferric-sodium EDTA, and DKW—Driver and Kuniyuki Walnut media.

## Data Availability

Data are contained within the article.
